# Building a rights-based approach to nutrition for women and children: harnessing the potential of women’s groups and rights-based organizations in South Asia

**DOI:** 10.3389/fpubh.2025.1461998

**Published:** 2025-02-05

**Authors:** Monica Shrivastav, Vani Sethi, Avishek Hazra, Zivai Murira, Roopal Jyoti Singh, Krishna Wagh, Anuradha Nair, Veronica Kamanga Njikho, Sapna Desai

**Affiliations:** ^1^PopulationCouncil Consulting, Noida, Uttar Pradesh, India; ^2^UNICEF Regional Office for South Asia, Kathmandu, Nepal; ^3^Population Council Institute, India Habitat Centre, New Delhi, India

**Keywords:** women’s groups, women’s rights-based organization, nutrition, rights, pathways, South Asia

## Abstract

Women face numerous gender-based barriers that hinder their access to resources, nutritious foods, nutrition services, and maternity entitlements. Evidence shows that certain types of women’s groups can improve women’s access to resources and social capital and in some approaches also improve health and nutrition outcomes. Women’s rights-based organizations in South Asia have a longstanding tradition of collective action toward gender equality. Women’s rights-based organizations work in areas such as microfinance, livelihoods, women’s rights, health, and combating violence against women. In this perspective article, we explore how women’s groups and rights-based organizations can leverage their collective strength to advance nutrition outcomes for women and children. We identify seven pathways implemented through women’s groups toward improving nutrition outcomes. These pathways include (i) income generation, (ii) agriculture, (iii) health and nutrition behavior change communication and participatory learning and action, (iv) advocating for rights to better health and social services, (v) food access, (vi) cash transfers, and (vii) strengthening service delivery and fostering convergence with health systems. We also note that women’s groups have the potential to implement integrated interventions through combined food-systems-rights pathways. Investing in this area can support transforming nutrition policy from a service delivery model to a rights-based approach.

## Introduction

South Asia accounts for 40 percent of the global burden of low birth weight (<2,500 gm) children (1), a barometer for women’s poor nutrition status before and during pregnancy. In the region, one in five women and adolescent girls are underweight and one in two are anemic (1). Adolescent girls and women who belong to the poorest wealth quintiles, have limited education, and reside in rural areas experience higher rates of underweight, anemia, and short stature (1, 2). Many women in South Asia have sub-optimal dietary practices and poor access to health and nutritional services, including antenatal care and iron supplementation, with geographic variation (2). Maternal nutritional outcomes are influenced by health system barriers that limit access to services. These include barriers related to frontline workers, such as vacancies, poor mentoring or motivation, increased work catchment load on existing health workers, and constraints in reaching distinct geographies (3–5). Discriminatory gender norms affect women’s decision-making power and autonomy, which in turn can contribute to adverse birth outcomes (6, 7).

Women’s groups are a common feature in most countries in South Asia (8). Women’s groups are generally defined as a group of individual women from a community who come together toward a common purpose. These include economic groups such as self-help or livelihoods groups, collectives formed with social action, health, and empowerment objectives, community-based women’s groups that work broadly on development objectives, or special population groups such as sex workers or new mothers’ groups (9). Additionally, women’s rights-based organizations have a long history in social and political movements in the South Asian region (10). These organizations are characterized by a collective pursuit of advancing women’s rights in a range of domains, with achievements such as improving women’s representation in political spaces (11), education, public and private safety, and gender-sensitive poverty alleviation (12). While there is some overlap, we have found that women’s groups often implement localized and community-based interventions, whereas women’s rights-based organizations work on a large scale.

Evidence from experimental studies indicates that membership in women’s groups can improve women’s financial inclusion, control over income, decision-making, and political participation (13–17), depending on specific design and implementation characteristics. Systematic reviews have also reported on the effectiveness of specific types of women’s groups and approaches in reducing maternal and neonatal mortality (18) and improving perinatal and child health practices (19). Different types of nutrition interventions have been implemented and evaluated via women’s groups to improve the nutritional outcomes of women and children. A 2018 systematic review by Kumar et al., summarizing 36 studies across Bangladesh, India, Nepal, and Pakistan, noted the potential of women’s groups in improving some nutrition-related practices but also highlighted variations in outcomes across different types of intervention pathways (20). Kumar et al. developed a framework to describe four pathways through which women’s group interventions can improve nutrition outcomes among women and children in South Asia, which include (i) income, (ii) agriculture, (iii) health and nutrition behavior change communication, and participatory learning and action, and (iv) rights (20). A subsequent 2020 systematic review in India found mixed evidence on the effectiveness of women’s groups in improving health and nutrition outcomes, with participatory approaches most commonly reporting positive effects. The review highlighted the importance of understanding which approaches work, where, and for whom (19).

Nutrition outcomes are complex and challenging to achieve for multiple reasons. One, nutrition practices, such as iron folic acid consumption, dietary diversity, and exclusive breastfeeding, require sustained inputs and support (21). Two, the effects of optimal nutrition practices are not seen immediately on measurable long-term outcomes, such as anemia, thinness, overweight/obesity, stunting, and wasting. And three, negative influences of other social determinants including but not limited to food accessibility/affordability, gender stereotypes, and prevailing socio-cultural norms. These factors highlight the need for a multidimensional approach and pathways to improve nutrition outcomes among adolescent girls and women. In this perspective, based on our experience as researchers, practitioners, and donors, and drawing from a recent scoping exercise in South Asia, we explore ways to harness the potential of women’s groups and rights-based organizations toward improved nutrition in South Asia.

## Expanding ways through which women’s groups intervention can improve nutrition outcomes

### Expand intervention pathways

**Figure 1:**
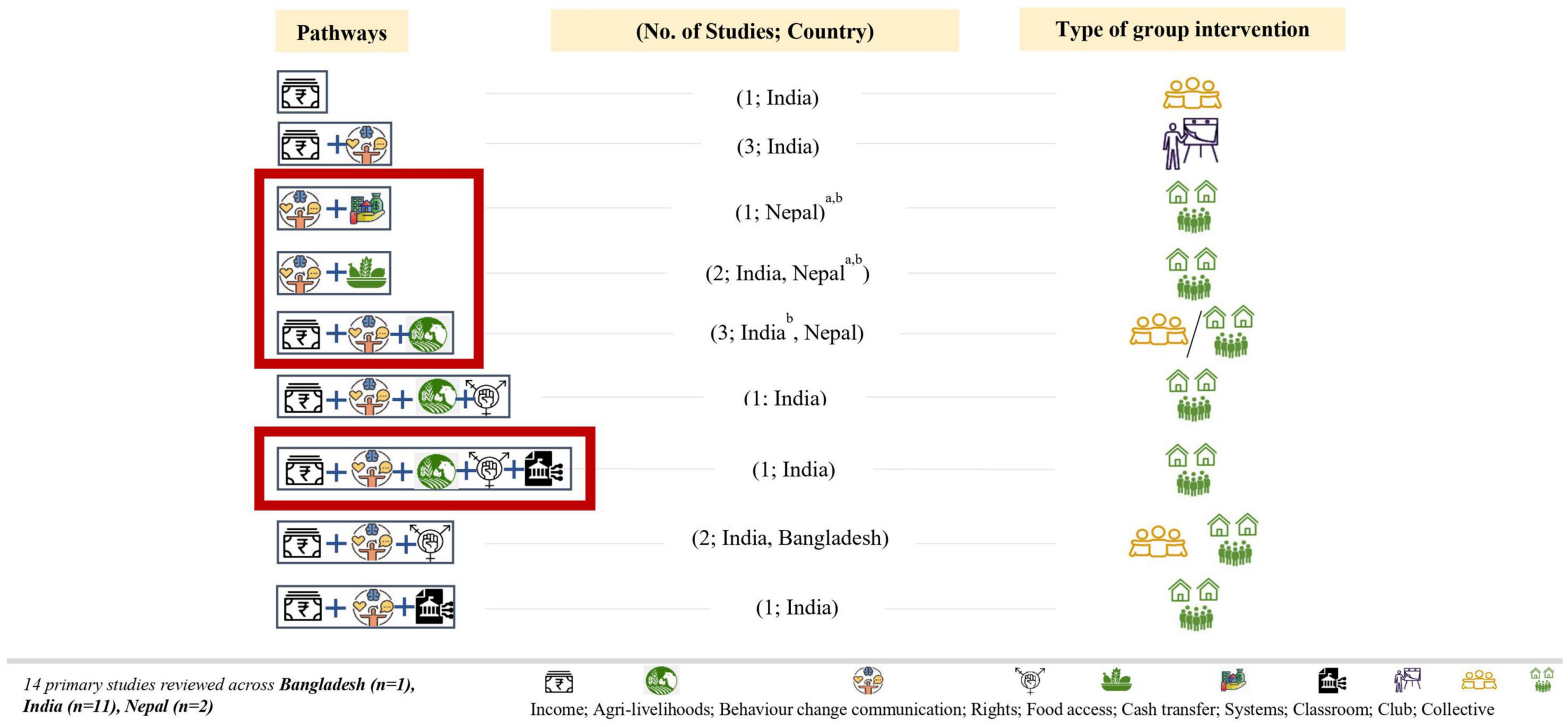
Women’s groups and nutrition: Intervention pathways. Source: Rapid review of literature by the authors. Note: Total 17 studies, including 14 primary and 3 process studies related to primary studies; ^a^One study in Nepal has two arms—cash and food transfer—for identifying the pathways, the authors have separated this study into two distinct pathways; ^b^Three studies (41–43) are shown as they were process/qualitative evaluations of the two studies (30, 34).

Building on Kumar’s 2018 review that noted four pathways through which women’s groups interventions can improve nutrition outcomes among women and children in South Asia, we synthesized 17 more recent studies and identified three additional pathways adopted by women’s groups: (i) food access, (ii) cash transfers, and (iii) strengthening service delivery and fostering convergence with health systems (22). In practice, many interventions employed combinations of the seven pathways to improve nutrition outcomes for adolescent girls, women, and children (Figure 1).

For example, in India, most interventions (21, 23–32) implemented multiple pathways through women’s groups, while only one (33) focused solely on the income pathway. One intervention utilized women’s self-help groups to implement five pathways: income, agriculture and livelihoods, participatory learning and action, rights, and system strengthening, aiming to improve nutrition outcomes for adolescent girls, pregnant women, and mothers of children under two (27, 28). Another experiment combined participatory learning and action with food provisions for young children via crèches to improve birthweight and child anthropometric measures (23). Some interventions integrated participatory learning and action with agriculture, livelihoods, and rights-based interventions to enhance maternal and child nutrition outcomes, all through open participation and collective approaches. Other combinations of pathways implemented via women’s groups included behavior change communication with either agriculture and livelihoods; social accountability actions; or health systems strengthening. Some interventions also focused on savings and credits through women’s self-help groups to enhance health and well-being. Similarly, in Nepal, the combination of pathways includes participatory learning and action with food transfers and cash transfers for pregnant women to improve birthweight and child anthropometric measures (34). Another experiment in Nepal combined behavior change communication using traditional health education interventions with agriculture and livelihoods to improve child growth and diet (35). An intervention in Bangladesh applied behavior change communication combined with social accountability actions for improving child nutrition outcomes (36).

### Tap the potential of women’s rights-based organizations

Women’s rights-based organizations in South Asia address a broad spectrum of issues, including microfinance, agriculture and livelihoods, natural resources and land rights, violence against women and girls, sexual and reproductive health rights, human rights and social justice, child rights, gender and security, skill development, political representation, and advocacy for special groups like home-based workers, sex workers and the LGBTQI community. Their geographic spread includes subnational, national and regional organizations.

**Figure 2:**
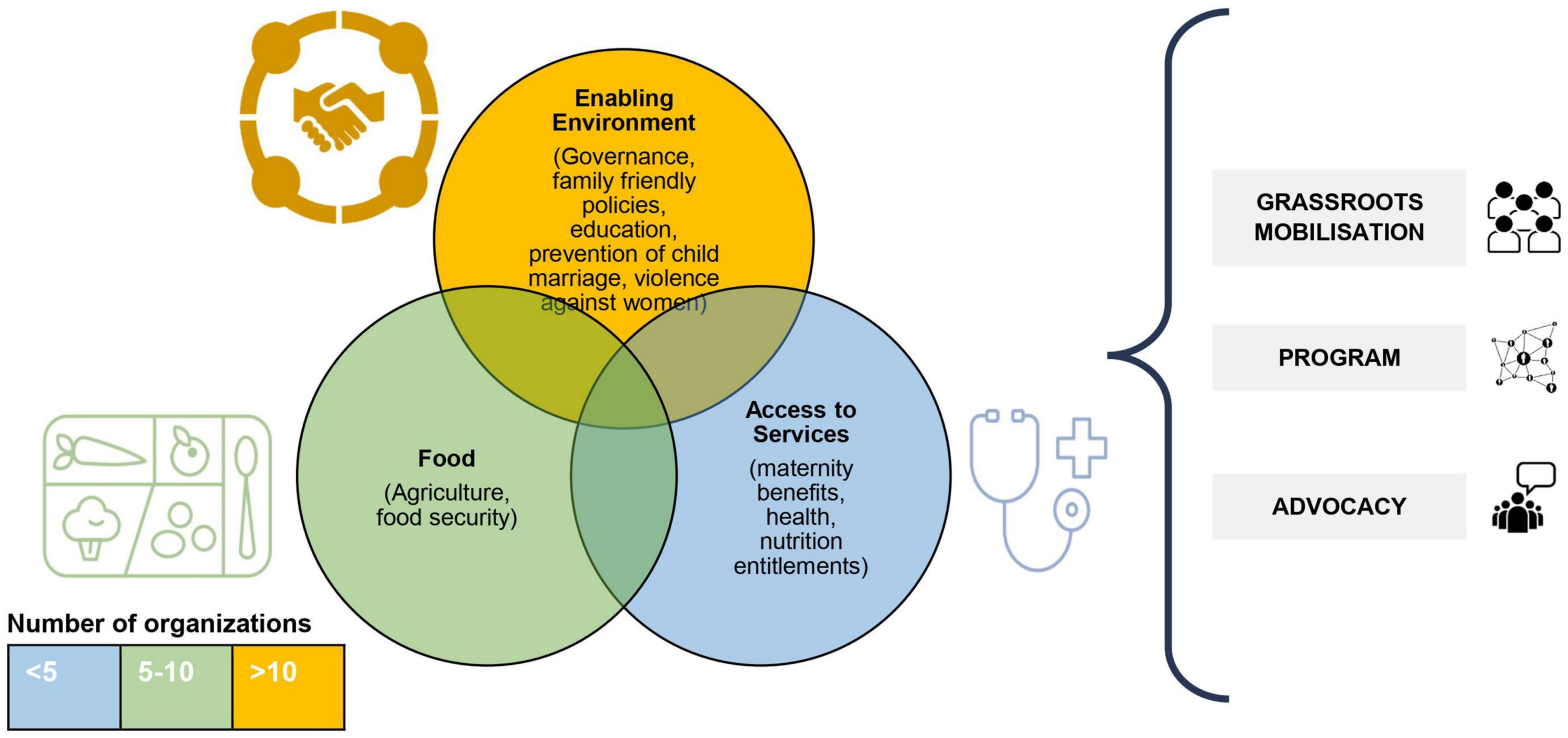
Approaches and actions used by women’s rights-based organizations.

We conducted a programmatic scoping review for the UNICEF Regional Office of South Asia from February to March 2023, engaging 20 organizations and seven experts in six countries: Afghanistan, Bangladesh, India, Nepal, Pakistan, and Sri Lanka. The scoping review aimed to explore the potential of engaging women’s rights-based organizations to improve women’s nutritional outcomes in South Asian countries. We learned that the women’s rights-based organizations act at three levels: mobilizing women at the community and grassroots level, through implementing programs, and by advocating for policy changes. They broadly focus on three intervention domains—(i) creating an enabling environment (governance, family-friendly policies, education, preventing child marriage, violence against women and girls), (ii) access to food (agriculture, food security), and (iii) access to services (maternity benefits, health, nutrition entitlements) (Figure 2).

Overall, 14 (of the 20) women’s rights-based organizations we spoke with across Afghanistan, Bangladesh, India, and Nepal work on creating an enabling environment by adopting multi-pronged strategies. These include campaigns, peer support, and leadership development to empower women as change-makers. Some organizations in Bangladesh and India use social audits and participatory methods to identify and prioritize issues for collective action. They build alliances with other networks and local groups to address a wide range of issues including land rights, livelihoods, and social issues like alcohol abuse and domestic violence. An organization in Nepal adopts the approach of establishing dialogs with communities and governments, they aim to strengthen governance and promote inclusivity. They also engage community-based women’s groups or networks to function as first responders or reporters of domestic violence cases and provide mental health support and psychosocial counseling. They undertake capacity building of local governments in addressing violence against women and facilitate dialogs with women’s groups. Organizations in Afghanistan establish special groups to support marginalized women, providing essential services like shelter, childcare, and vocational training. A rights-based organization in Bangladesh has made efforts to advocate and strengthen the gender-responsiveness of government systems to address violence against women. Among these efforts are lobbying for better services and accountability within the system, mobilizing women’s constituencies, engaging local government and constituencies for improved governance, and advocating for women’s issues and redressal through increased women’s representation at ward levels and forums.

We identified eight organizations from those we spoke with who were working to improve food access and agricultural practices. Some organizations in Afghanistan, India, and Nepal aim to improve access to natural resources as a right for the communities to ensure local availability of food, and access to resources, and promote local food production and consumption as well as food preservation. They organize women at the village level, arrange public hearings, and engage in campaigns. Interventions such as nutrition-sensitive agriculture methods are implemented to diversify food production for small-landholding and landless farmers. These interventions also promote local or indigenous food consumption to improve women’s decision-making control over production, selling, and consumption, thereby linking ‘field to plate’. Other interventions by organizations in Bangladesh, include setting up micro-enterprises, establishing market linkage and food fortification and processing units, community cooking, and creating grain and seed banks. These approaches aim to enhance livelihoods, agricultural practices, and household food security.

Five women’s rights-based organizations in Afghanistan, Bangladesh, India, Nepal, and Pakistan adopt strategies to facilitate dialog between community and government systems to bridge the gap and build synergy to improve community access to health services and entitlements. For example, organizations in Bangladesh, India, and Nepal implement group-led activities, including health camps with local government, nutrition rehabilitation centers, and referral/ linkage to health services to facilitate access to reproductive health and nutrition rights. Organizations in humanitarian settings (Afghanistan) undertake activities to establish private clinics to provide primary healthcare support to women as their right. Efforts are also made to strengthen state institutions by providing technical assistance for capacity building, planning, and monitoring of interventions. An organization in Pakistan advocates for representation from women’s organizations at forums and committees. It adopts a bottom-up approach to facilitate dialogs between women from communities and the government systems to address their demands and improve access, coverage, and quality of services, rights, and entitlements.

In addition, we found strategies in which local mobilizers with women’s self-help groups implement actions at the community level to address gender norms and practices, create awareness, generate demand for services, and support access to rights. Organizations also foster multi- and inter-departmental convergence to improve coverage, intensity, and quality of services. Other methods implemented to improve access to maternity entitlements and rights for the unorganized rural sector include influencing acts, public hearings, networks and campaigns, and community-level monitoring of outcomes.

## Priorities for investment and exploration

Partnering with women’s groups and rights-based organizations offers an opportunity to invest in nutritional outcomes, a long-neglected area of women’s rights. Women’s group interventions have demonstrated the possibility of adopting a multi-sectoral approach to improve nutrition and health outcomes in South Asia. While evidence on their effectiveness is mixed, it is clear that interventions are more likely to improve nutrition outcomes through adopting multi-pronged, participatory strategies with adequate intervention intensity (19, 37).

Moreover, social determinants play a critical role in impacting nutritional outcomes. For example, gender inequality adversely affects the nutritional status of women and children (38, 39). We argue that to address the social determinants of poor health and nutritional outcomes, a rights-based approach is critical—moving beyond service delivery interventions. Investments need to be expanded in South Asia to ensure a key role for women’s organizations and to experiment with various strategies, such as harnessing a combined food-systems-rights pathway and integrating participatory approaches to improve nutritional outcomes. However, improving such outcomes will require continuous, intensive, and sustained actions at scale (40). Any future experiments should consider a longer intervention duration, ensuring adequate program implementation intensity at scale and building capacities of communities for a sustained impact. It is critical to ensure local, women-led leadership to the spirit of a ‘movement’ grounded in rights approach rather than only service delivery or add-on interventions that use women’s groups as a delivery mechanism.

Across countries, we propose that the next step could be the development of a network in South Asia that brings together organizations/ institutions to collaborate on a shared rights-based nutrition agenda. A network of organizations that collaborate and exchange knowledge and practices through convening’s, joint research, and policy action. Moreover, joint activities can strengthen advocacy to ensure a key role for women’s rights-based organizations in addressing nutritional outcomes across the region.

## Conclusion

Women’s groups and organizations are strategically placed to address nutrition through collective and group-level action. Working with women’s groups and rights-based organizations can ensure justice and equity in nutrition agendas, where women are at the forefront of deciding their priorities, demanding their rights and services, and acting and pushing for social change and accountability through collective action. Such partnerships can support transforming nutrition programs from a service delivery to a rights-based approach.

## Data Availability

The original contributions presented in the study are included in the article/supplementary material, further inquiries can be directed to the corresponding author.
